# Predicting academic achievement and student absences in high school: The roles of student and school attributes

**DOI:** 10.3389/fpsyg.2023.987127

**Published:** 2023-03-28

**Authors:** Georgios Sideridis, Abeer A. Alamri

**Affiliations:** ^1^Boston Children’s Hospital, Harvard Medical School, Boston, MA, United States; ^2^National and Kapodistrian University of Athens, Athens, Greece; ^3^Education and Training Evaluation Commission (ETEC), Riyadh, Saudi Arabia

**Keywords:** academic achievement, school absences, school resources, parental education, multilevel modeling

## Abstract

The present study aims at examining predictors of high school students’ academic achievement from student-level and school-level predictors in the Kingdom of Saudi Arabia, especially in light of policy mandates on educational reform in accordance with Vision 2030. Participants were 528,854 individuals who took on the Standard Achievement Admission Test (SAAT), along with other demographic variables. The mean age of participants was 19.7 years with an *SD* = 1.87. There were 234,813 males and 294,041 females. A Multilevel Random Coefficient Modeling (MRCM) model was engaged to identify predictors of academic achievement. Results indicated the positive roles of being a female, having educational parents, being educated in religious schools or large schools, and having small student-to-teacher ratios and the negative roles of student absences, student age, and being educated in new schools. Results are viewed under the lenses of new policy mandates on educational reform in the Kingdom of Saudi Arabia.

## 1. Introduction

“Education is invaluable for creating a better world by promoting the values of a culture of peace, mutual understanding and international solidarity, and its achievements in this regard denote its quality” (Singh, 2012, p. 6). Quality education resulting in high achievement gains is then the ultimate goal of education. However, concerns have been raised that basic education is of low quality. The Global Compact on Learning initiative (2011) stated that “Beyond the 67 million children who are not attending primary school in low-income countries, there are countless children who are going through 5 years of education without learning basic reading, writing, and math skills.” Consequently, several bodies have expressed their views on providing the right to quality education for all students. The merging of policymaking and research is then imperative for improving the means and for the provision of quality education for all. Policymakers are then in the need of identifying important determinants of students’ achievement that have been validated in research. The present study focuses on identifying predictors of achievement in high school that span both the student level (such as gender, age but also the number of absences students made, and parents’ education, etc.) but also the school level (e.g., private versus public or religious versus non-religious schools, older versus newer establishments, school resources, and school size).

The selection of the Saudi Arabia Kingdom warrants attention as several socio-cultural, ethnic and economic reasons distinguishes the Kingdom from the rest of the world. Specific cultural differences include segregation across gender during schooling, academic differences in achievement favoring females, the operation of a culture of night studying, the emittance of a large number of absences from school, religious considerations, and other (Baki, 2004; Hamdan, 2005; Khan, 2011; Al-adwan and Smedley, 2012; Hammad and Shah, 2018). Specifically, based on TIMSS data in 2019, and both cohorts, the academic performance of Saudi students ranged 53 among 58 countries thus reflecting significantly lower achievement levels in mathematics, language and science. In science 46% of the Saudi students achieved basic levels compared to almost 100% in Asian countries such as China and South Korea and non-Asian countries such as Russia. In language, pre-literacy activities at home showed a positive relationship to subsequent language skills suggesting the need to look at parental practices at home. In contrast and despite the relatively low achievement outcomes, parents in Saudi Arabia reported being significantly more satisfied with students’ achievement in school (80%) compared to the international average of 64%. Furthermore, although discipline problems were lower in Saudi Arabia, the documented negative relationship between discipline and achievement was not observed in the Kingdom. With regard to resources, there was limited access of computers during math (31% of the classes) compared to, e.g., 90% of the classes in Malta, New Zealand, and Denmark. Last, student absences have also been linked to lower mathematics performance and in Saudi Arabia the number of absences was much large (28%) compared to the international average of 11%. Related to the emerging culture of nigh studying and/or sleep deprivation (Gaultney, 2010; El Hangouche et al., 2018; Zeb et al., 2020), 29% of the Saudi students reported feeling tired every day and comparatively their science scores were lower than 40 points. The above empirical findings point to the need to investigate further the role of these factors. As the TIMSS 2019 Report reported: “These results are alarming and indicate that a considerable number of students in Saudi Arabia do not have the basic knowledge of mathematics and science. These students will not only be unable to continue education, but their limited knowledge will not allow them to fully participate in modern technology-rich society. Moreover, the shortage of students who excel in mathematics and science presents a challenge for the future of Saudi Arabia where the goal is to develop a knowledge-driven economy” (p. 12, TIMSS 2019 Report, 2021).

### 1.1. Academic achievement – Theoretical and empirical considerations

Academic achievement may be defined as the accomplished success in any educational task or an individual’s ability to reach a set goal through effort, skill, or courage within the school context (Hornby, 2006). Academic achievement is a critical factor in students’ future academic and professional careers since it is usually closely linked to successful university entrance or job success. Academically successful individuals are more likely to be accepted to more prestigious educational institutions, have better employment opportunities, earn a high income, and experience high life satisfaction (Liu et al., 2020). Traditionally, two different indicators are used to determine academic achievement: high school grades (or Grade Average Point – GPA) and standardized achievement tests (e.g., SAT, GRE, GMAT, PISA, TIMSS, etc.).

Several studies show that GPA may not always accurately capture students’ performance, mainly due to a phenomenon known as *grade inflation*. There are several reasons why grade inflation exists. First, high schools attempting to climb over the school ranking ladder deliver high grades to their students; this practice is linked to school reputation enhancement and is associated with increased school income by attracting more and better-qualified students. Second, teachers’ provision of high grades is an indicator of their students’ performance and hence, their efficacy as instructors (Swift et al., 2013). On the other hand, standardized achievement measures are designed to assess the degree of knowledge and/or skill acquisition, attributed to classroom instruction (Airasian and Russell, 2008). They are norm-referenced and engage standards and norms for administration and scoring. Everyone taking the test receives the same directions, has the same restrictions of time and resources, and his/her response on the items is scored based on pre-set objective criteria. Finally, the interpretation of the scores is not dependent on contextual variables (e.g., school quality, teachers’ qualifications), but rather on the students’ relative information about their readiness to undertake university coursework (Phelps, 2005; Benbassat and Baumal, 2007).

Most achievement tests assess some combination of verbal, quantitative, writing, and analytical reasoning skills. The combination of these skills is a necessary condition for successful degree completion in most fields of study (Kuncel et al., 2010). A considerable amount of empirical evidence has shown that standardized test scores are robust predictors of college success (e.g., Noble and Sawyer, 2002). Camara and Echternacht (2000), reanalyzing data from others’ studies, demonstrated that the correlation between SAT scores and college success ranged from 0.49 for African American and Hispanic males to 0.63 for Asian American males. Additionally, Kuncel et al. (2010) found that both Graduate Record Examination (GRE) Verbal and Quantitative scores were robust predictors of first-year graduate GPA and GPA after graduation in both masters and doctoral programs.

### 1.2. Theoretical model of the present study

**Figure 1:**
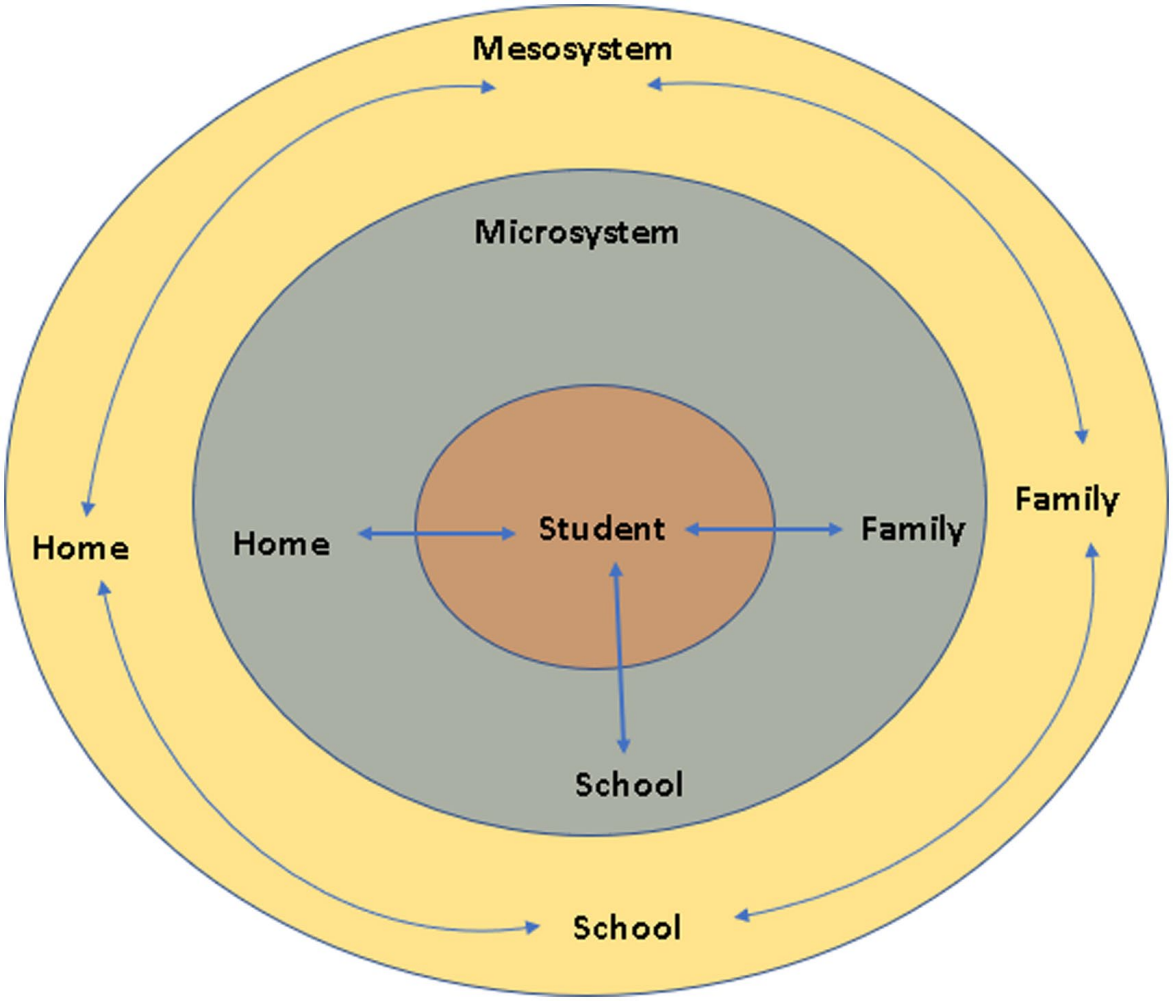
Hypothesized ecological model utilized in the present study with three layers.

The present study was guided by the ecological model of Bronfenbrenner (1979) in that the relationship between variables among different ecological layers interact with each other in a reciprocal and bidirectional manner to explain children’s behavior. In other words, as Martin and Lazendic (2018) stated: “diverse processes in a student’s educational ecology influence his/her educational outcomes” (p. 466). Within this ecological framework then, achievement is determined by the contextual variables that provide supports for the individual tendencies to grow and develop (Bronfenbrenner, 1992, 2001). The educational ecologies explored in the present study were (a) student level factors (e.g., gender, age, number of absences), (b) home factors (e.g., parental education), and (c) school factors such as amount of school resources, type of school, and student teacher ratio. These ecological factors were expected to exert direct influences on academic achievement. The ecological framework evaluates the contribution of variables at different levels where the educational processes play out. Such structures can only be evaluated with the use of multilevel models that take into account the hierarchical structure of the data, as students are nested within schools and intra-individual, individual, and contextual processes can be simultaneously evaluated (Gonzalez, 2009). Below there are empirical findings on the roles of individual, home, and contextual (school) factors. Figure 1 displays the three layers of Bronfenbrenner’s ecological systems theory utilized in the present study with the student interacting with the microsystem variables such as home, family and school and the mesosystem including interactions between the microsystem factors.

### 1.3. Student factors on academic achievement

#### 1.3.1. Gender and academic achievement

Several studies have shown that females perform better than males in various aspects of academic life (i.e., better grades, higher levels of motivation, better adaptation, etc.), establishing the so-called *gender gap* in educational attainment (e.g., Voyer and Voyer, 2014; Bugler et al., 2015). The reasons for these gender differences in school achievement have not been clarified yet, mainly due to the inconclusive findings. For example, several studies suggest that females outperform males in language-based subjects (Deary et al., 2007; Spinath et al., 2010), and males outperform their female counterparts in STEM-related subjects (e.g., math, engineering; Lakin, 2013). However, Voyer and Voyer (2014) found that females appear to have higher school grades in STEM subjects compared to males, and Else-Quest et al. (2010), in a meta-analytic study, showed negligible gender differences in the results of standardized math tests. Consequently, with regard to differences in achievement across gender, the jury is still out.

#### 1.3.2. The roles of student absences in academic achievement

Research evidence has been unequivocal in designating the negative role of absences in academic achievement (Balfanz and Byrnes, 2012; Gottfried, 2019; Kearney et al., 2019a,b) suggesting causal links. Direct effects have been revealed between absences and low achievement in reading and math (e.g., Chang and Romero, 2008; Moonie et al., 2008; Gottfried, 2010). Literally, all studies dealing with this subject have pointed to the negative roles of absences with regard to school achievement (Smerillo et al., 2018) regardless of being authorized or unauthorized (Havik et al., 2015; Skedgell and Kearney, 2016, 2018). The extent to which absences affect students’ lives has been evident in the work of Balfanz and Byrnes (2012) who estimated that severe absenteeism (termed chronic absenteeism) occurs between 5 and 7.5 million students in the United States. Chronic absenteeism has been inconsistently defined but the general agreement is around 15 days per school year (or ~15% of school time) but provides us with an important baseline value to evaluate the magnitude of the phenomenon in the high schools of the Kingdom of Saudi Arabia. Interestingly, student absences have not only been linked to low achievement but also with significant reductions in the probability of graduating (Smerillo et al., 2018), the probability of dropping out of school (Mac Iver and Messel, 2012; Schoeneberger, 2012) on maintaining positive motivational states and persistence (Balfanz and Byrnes, 2012) and on avoiding future academic difficulties (Schoeneberger, 2012). Nevertheless, students’ gender may moderate the relationship between absences and achievement as males emit more absences than females (Veenstra et al., 2010; Sälzer et al., 2012), and this is also an objective of the present study.

### 1.4. Home factors on student’s achievement

#### 1.4.1. The roles of parent education

There is long-standing evidence suggesting that parent education is important for students’ academic and professional success (e.g., Whiston and Keller, 2004; Davis-Kean, 2005). The pathways to linking parents’ education to their student’s academic achievement have been through family processes. For example, Dornbusch et al. (1987) suggested that parental education affects parenting style (i.e., more permissive and less strict in parenting), which is then linked to children’s academic achievement. Other studies have shown the mediating role of income and SES (Morrissey et al., 2014), parental expectations (Pinquart and Ebeling, 2020), parental engagement and involvement (Dubow et al., 2009), and many more.

Liang and Bogat (1994) reinforced the notion that parental educational attainment influences students’ achievement levels. They reported that students’ graduation and entry to university were linked to highly educated parents. Moreover, parents’ educational level may act as a protective factor with regard to academic dropouts (Blondal and Adalbjarnardottir, 2014). Concerning achievement on standardized measures, Zwick and Green (2007) reported a positive relationship between students’ SAT scores and parents’ educational (Machebe et al., 2017). For the above reasons, it would be important to replicate the positive effects of parents’ education on student achievement in Saudi Arabia, for which, little empirical evidence is available but also include parental education as a control variable so that every other coefficient will be adjusted accordingly, a practice implemented in past research (Van Hek et al., 2018).

### 1.5. School factors on student’s achievement

#### 1.5.1. The role of school resources

The approach taken in the present study is that all forms of school resources represent capital that in various forms serve specific educational aims. Empirical evidence has supported a quantitative approach in that “better resourced” schools have been linked to better achievement outcomes of their students (Sullivan et al., 2013), whereas those with a shortage of instructional materials, low quality facilities, and equipment with poor educational outcomes (Uline and Tschannen-Moran, 2008). For example, Gigliotti and Sorensen (2018) reported significant effect sizes in English and math as a function of increased school resources, which they translated to spending an additional $1,000 per student. They considered this amount to be small compared to the associated academic gains from that increase. Last, statistical evidence on the need to incorporate school resources comes from an empirical study by Aburizaizah et al. (2019) who reported that two thirds of the variability in student achievement scores lied in between school level variables, not among student level characteristics. In the present study, each specific variable manipulated within this context of school resources is discussed next.

#### 1.5.2. Student-to-teacher ratio

An important variable, the ratio of students to available teachers, has been a significant predictor of academic achievement (Blatchford and Lai, 2012) as overcrowded classrooms are likely to undermine quality educational practices. This ratio is considered an indicator of resource allocation in education. Several studies have reported significant benefits in small group instruction based on small student-to-teacher ratios (Hattie, 2005), although others have reported mixed results (Fredriksson et al., 2013), or no impact at all (Dearden et al., 2002). Others have reported no significant benefit from small student-teacher ratios (Hanushek, 1986, 1989), which they attributed to the use of standardized assessments. Interestingly, Graddy and Stevens (2005) found that although in state schools, the student-to-teacher ratio had little or no effect on achievement, there was a significantly positive relationship observed in private schools only. They attributed this finding to the low student-to-teacher ratio observed in private schools, which along with teacher qualities and individualized instruction students’ achievement gains are enhanced (Dearden et al., 2002).

**Figure 2:**
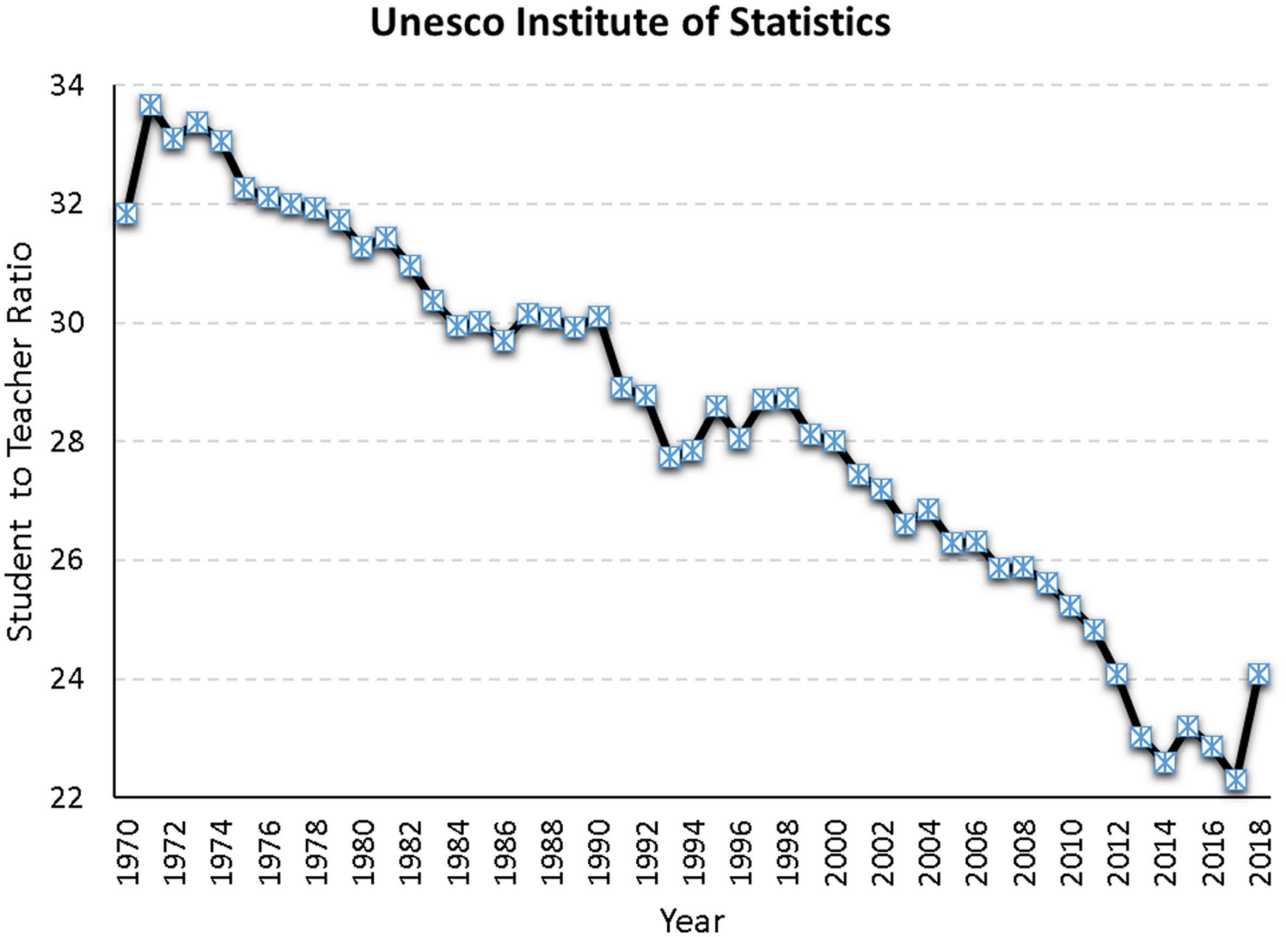
Ratio of Student to Teacher using world data from 1970 to 2018 (http://uis.unesco.org/).

Based on the 2008 Unesco report, the global average of the teacher to student ratio is 14 pupils per teacher (Unesco Institute for Statistics, 2008), although countries such as India have reported ratio averages of around 35 in secondary schools (Singh, 2012). This average had a range of 10.9–26.8. Figure 2 displays international estimates from the Unesco Institute for Statistics showing a negative trend over time in that ratios improve with time. The estimated ratio for Saudi Arabia has been 14. In a more recent report, the ratio of teachers to students in secondary education was 13.9 and the respective estimate in Saudi Arabia was 11.5, which is close to the global average (OECD, 2020), albeit a bit lower. Given the salient role of teacher-to-student ratios, it will be important to establish the predictive validity of the measure with national data in Saudi Arabia. This examination is particularly more relevant under the lenses of the Covid pandemic for which attendance is restricted mostly to distance learning means.

#### 1.5.3. School materials and resources

There is ample evidence that educational resources are positive correlates of academic achievement (Rivkin et al., 2005), although this research line is by far from conclusive. For example, Coleman et al. (1966) argued that schools have a minor influence on student achievement. Hanushek (1989, 1997) also found insufficient evidence regarding the positive role of school resources on students’ performance. On the other hand, Greenwald et al. (1996), using meta-analytic techniques, re-examined Hanushek’s evidence and recognized a weak, albeit significant positive relationship between school resources and student achievement. Similarly, Card and Krueger (1996) and Adeogun and Osifila (2008) found that school resources (e.g., rooms, labs, equipment, etc.) and economic resources (e.g., per-pupil expenditure) have a positive effect on students’ achievement. However, in a more recent meta-analytic study, Hedges et al. (2016) found no significant relationship between capital spending per pupil and academic achievement. Wößmann (2003) suggested that educational resources have a trivial effect on academic achievement, although Savasci and Tomul (2013), analyzing data from PISA exams in Turkey, concluded that the lack of physical resources has a harmful effect on student performance.

What complicates the role of school resources are two main things: (a) how the quantity of resources is related to school size for which non-linear relations have been observed, and (b) what constitutes the operational definition of resources. For example, the student-to-teacher ratio has been recently considered a resource, although it has not always been the case. Consequently, concerning the role of school resources, the jury is still out, and this question is also an important goal of the present study.

Thus, the present study aims at examining predictors of high school students’ academic achievement from student-level and school-level predictors in the Kingdom of Saudi Arabia, especially in light of policy mandates on educational reform in accordance with Vision 2030. The present findings would reflect on the mandates put forth by King Abdullah bin Abdul Aziz for the development of the public education project (KAAPEDP) established in 2010 which targeted teacher’s development of practical skills and theoretical knowledge to fulfill the new requirements and standards of teacher, along with emphasizing Islamic values and principles. Thus, the following research questions were posited:

1. How do student-level attributes such as gender, age, number of absences, and parents’ education predict high school achievement?
2. How do school-level characteristics such as private versus public, size, and available equipment predict students’ high school achievement?
3. What among student and school-level attributes predicts students’ absences?

## 2. Methods

### 2.1. Participants and procedures

Participants were 528,854 individuals who took on the Standard Achievement Admission Test (SAAT), along with other demographic variables. Data became available after merging databases with information coming from the Educational Testing and Evaluation Committee (ETEC) and the Ministry of Education of the Kingdom of Saudi Arabia. The mean age of participants was 19.56 years with an *SD* = 2.07. There were 234,813 males and 294,041 females. Data were collected within the 2019–2020 academic period.

### 2.2. Measures

#### 2.2.1. Standard Achievement Admission Test

Standard Achievement Admission Test is an admission test that assesses four scientific domains namely biology, chemistry, physics, and math. SAAT is administered by the National Center for Assessment (NCA) in Riyadh, Kingdom of Saudi Arabia, and tested the general and key concepts covered in the last three grades of General Secondary School as follows: 20% of each subject for the first year of the high school syllabus, 30% of each subject for the second year of the high school syllabus, and 50% of each subject for the third year of the high school syllabus. SAAT is comprised of 88 multiple-choice items, and the test time for each section is 25 min. Items utilize a dichotomous scaling format. The total score of the SAAT was used in the present study. Validity studies of the measure have been reported earlier (Dimitrov et al., 2015). Other data collected involved the number of student absences, parents’ educational level, school type (private, religious, etc.), number of students and teachers in the school, year in which school was established, and the presence of computers and other science labs. These variables were all included in the prediction of student achievement from both student-level and school-level variables. Variables excluded from the analyses involved several terms that were collinear. For example, the number of teachers in the school was collinear with the number of Saudi teachers and/or new teachers. Consequently, only the number of teachers was employed, regardless of origin, from which the ratio of student to teacher was estimated. Table 1 displays the demographic information of the measured variables.

**Table 1:** Descriptive statistics of variables employed in the present study.

Variables	Mean/Freq.	SD	Min	Max
SAAT	66.07	13.359	30	100
Student Absences	15.2	14.151	0	141
Females	294,041 (55.6%)			
Males	234,813 (44.4%)			
Age	19.56	2.066	17	26
Father’s education	6,138 (7.7%)			
Illiterate	6,824 (8.5%)			
Literate-read and write	10,272 (12.8%)			
Elementary school	13,671 (17.1%)			
Intermediate school	21,537 (26.9%)			
High school	20,030 (25%)			
College	1,137 (1.4%)			
Masters	572 (0.7%)			
Doctorate				
Mother’s education	12,210 (15.1%)			
Illiterate	8,223 (10.2%)			
Literate-read and write	13,554 (16.8%)			
Elementary school	12,641 (15.7%)			
Intermediate school	17,585 (21.8%)			
High school	16,035 (19.9%)			
College	302 (0.4%)			
Masters	153 (0.2%)			
Doctorate				
Religious school	167 (3.1%)			
No of classes	7.72	4.77	1	72
No of admin	3.87	4.917	1	33
New schools (year of establishment mode value)	1995 (1416ad)		1 year	67 years
Lab on computers	2	0.81	0	56
Lab on physics	0	0.4	0	15
Ratio student/teacher	141	10	1	645

### 2.3. Data analyses

Data were analyzed using Multilevel Random Coefficient Modeling (MRCM; Raudenbush and Bryk, 2002). Initially, an unconditional or null model (Nezlek, 2001) was fit to the data to test whether prerequisites to multilevel modeling assumptions were met. These involved estimating the intra-class correlation coefficient, the design of effect index, and the reliability or separation of level 1 (student) units. When two-level structured data are treated in a single level, the total variance/covariance is used as a whole. This would violate the traditional independence of the unit of analysis assumption. However, when multilevel models are used, this violation will be recognized and the interdependence of the data will be accounted for (Raudenbush and Bryk, 2002). The Intra-Class Correlation (ICC) coefficient (Raudenbush and Bryk, 2002; Maas and Hox, 2005) was utilized to test for the presence of variability at each level in the analysis. The coefficient is estimated as the ratio of the between-level variance $\sigma_{u0}^{2}$ to that of the total variance (within $\sigma_{r}^{2}$ and between $\sigma_{u0}^{2}$ ). Although there is no rule of thumb for the ICC value, an ICC > 0.05 is considered substantial. Table 2 displays, ICCs for the two dependent variables, with the lowest coefficient being >21%, justifying the use of multilevel modeling procedures. Second, we estimated the “design effect” index (Muthen and Satorra, 1995) as a means of correcting the negative bias associated with nested data due to the violation of the independence of standard errors assumption. Third, we estimated the reliability (separation) of level-1 units with values below 0.10 being suggestive of using grand slopes. These results are shown in Table 2. The following model was fit to the data to predict high school achievement from student-level and school-level predictors, with the clustering variable being school:

**Table 2:** Prerequisites to multilevel modeling with random effects.

Dependent variable	T_00_	L-1 Var.	ICC	DEFF	Reliability
SAAT total score	51.156	120.118	0.299	29.673	0.951
Number of student absences	16.032	58.011	0.217	21.786	0.929

**Level-1 Model**


$$\begin{array}{l} SAAT_{ij}=\beta_{0j}+\beta_{1j}{}^{*}\left(Gender_{ij}\right)\\ \;\;+\beta_{2j}{}^{*}\left(AGE_{ij}\right)\\ \;\;+\beta_{3j}{}^{*}\left(No\;ofAbsences_{ij}\right)\\ \;\;+\beta_{4j}{}^{*}\left(Father^{’}s\;Education_{ij}\right)\\ \;\;+\beta_{5j}{}^{*}\left(Mother^{’}s\;Education_{ij}\right)+r_{ij} \end{array}$$

**Level-2 Model**


$$\begin{array}{l} \beta_{0j}=\gamma_{00}+\gamma_{01}{}^{*}\left(Religious_{j}\right)\\ \;+\gamma_{02}{}^{*}\left(No\;ofClasses_{j}\right)\\ \;+\gamma_{03}{}^{*}\left(No\;ofAdministrators_{j}\right)\\ \;+\gamma_{04}{}^{*}\left(Age\;ofSchool_{j}\right)\\ \;+\gamma_{05}{}^{*}\left(No\;of\;ComputerLabs_{j}\right)\\ \;+\gamma_{06}{}^{*}\left(No\;of\;PhysicsLabs_{j}\right)\\ \;+\gamma_{07}{}^{*}\left(RatioofStudent/Teacher_{j}\right)+u_{0j} \end{array}$$

$$\beta_{1j}=\gamma_{10}+u_{1j}$$

$$\beta_{2j}=\gamma_{20}+u_{2j}$$

$$\beta_{3j}=\gamma_{30}+u_{3j}$$

$$\beta_{4j}=\gamma_{40}+u_{4j}$$

$$\beta_{5j}=\gamma_{50}+u_{5j}$$

with *i* denoting a student nested in *j* schools. The term β_0j_ reflects the intercept term for SAAT. The terms *γ_01−_γ_08_* reflect partial regression coefficients at the school level. The terms β_j_ reflect partial regression coefficients for the prediction of SAAT scores from school-level predictors. The terms r_ij_ and *u_0j_* reflect residual variations around students and schools, respectively. It is also important to note here, that not all variables were included in the final model. An iterative procedure took place so that collinear predictors were removed from further consideration such as the number of teachers and the number of Saudi or international origin ones.

## 3. Results

### 3.1. Prerequisite analyses to employing a nested structure

Table 2 shows the results from the prerequisite analyses with the three last column findings corroborating with the idea that modeling random effects was the proper choice with these data. Specifically, the ICCs suggested the existence of large amounts of variability at both levels in the analyses and consequently the presence of a correlated structure that would be associated with smaller (and consequently biased) standard errors if modeled *via* regression analysis. The amount of variability in the data that were at the school level (and warranted the need to include school as a random variable) ranged between 18.4% and 29.9% which is both significant and substantial. The design effect estimates were also >2, an earlier recommended cutoff level as recommended by Muthen and Satorra (1995). Last, reliability was high, over 0.900 across all dependent variables suggesting that students were randomly varying over schools and the utilized multilevel approach should be implemented.

### 3.2. Dependent variable: High school achievement

#### 3.2.1. Unconditional model

**Figure 3:**
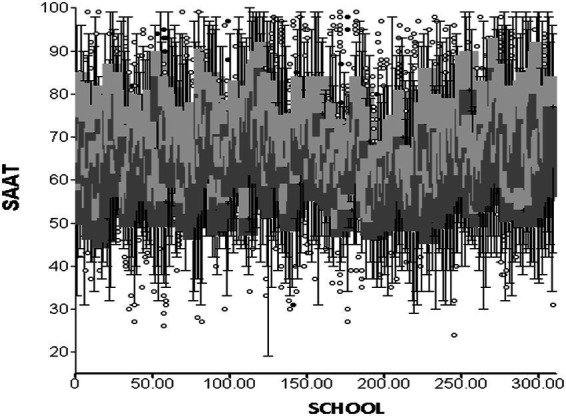
Variability of schools across SAAT scores; figure displays boxplots for a random. Number of schools (10% of them for clarity).

Table 2 shows estimates of variability at the student and school levels. Using the intra-class correlation coefficient estimates of ICC was 29.9% for the total achievement score. The respective estimate for the number of student absences was 21.7%. Consequently, there was a need to model the data accounting for the nesting of students within their respective classes. A visual view of the variability of the school’s performance is shown in Figure 3 with boxplot estimates across a random number of schools. Subsequent results display the effects of student-level and school-level predictors on SAAT achievement scores using a stepwise procedure.

#### 3.2.2. Level-1 predictors

Table 3 displays the findings from adding one prediction at each level of the analysis. Results indicated that the inclusion of gender was associated with a significant improvement in model fit using the difference in deviance estimates (improvement of 799 chi-square units). Given the coding of the gender variable, females had significantly higher SAAT total scores compared to males, all else held constant (Model 2). When age was factored in the model the gender effect remained significant as previously and a negative significant coefficient (−0.698) described the effects of age with older students being associated with significant decrements in SAAT total scores (Model 3). Again, the inclusion of age was associated with a significant improvement in model fit. In Model 4, the number of absences was added in the model, uncentered, leading to again a significant improvement in model fit. Results indicated that the more the absences the lower the achievement on the SAAT (by a rate of −0.266 in SAAT scores per absence). Models 5 and 6 included the effects of parents’ education. Results pointed to significant enhancements in SAAT total scores for one unit of change in parents’ education (categorically ordered variable). Interestingly, the effects of a father’s education were more pronounced compared to those of mothers, and that effect was confirmed using a chi-square difference test [*χ*^2^(1) = 12.704, *p* < 0.001]. Again, model fit was improved by a significant margin using the deviance statistic.

**Table 3:** High school achievement prediction from student level and school level variables.

**Parameter**	**Model1**	**Model2**	**Model3**	**Model4**	**Model5**	**Model6**	**Model7**	**Model8**	**Model9**	**Model10**	**Model11**	**Model12**	**Model13**
Fixed Effects													
Intercept	65.741*	61.234*	74.946*	82.149*	81.699*	79.480*	79.516*	78.082*	78.374*	78.693*	78.707*	77.037*	77.725*
Student level predictor													
Females		6.916*	6.953*	10.920*	8.811*	8.718*	8.474*	8.823*	7.936*	7.940*	7.958*	8.203*	8.277*
Age			−0.698*	−1.241*	−1.280*	−1.190*	−1.191*	−1.244*	−1.236*	−1.253*	−1.253*	−1.174*	−1.163*
No of absences				−0.266*	−0.260*	−0.255*	−0.255*	−0.255*	−0.255*	−0.255*	−0.255*	−0.254	−0.253*
Father’s education					1.194*	0.840*	0.838*	0.832*	0.830*	0.830*	0.830*	0.830*	0.830*
Mother’s education						0.633*	0.633*	0.620*	0.619*	0.618*	0.618*	0.620*	0.618*
School level predictor													
Religious school							5.699*	6.687*	6.733*	6.771*	6.762*	6.935*	6.590*
No of classes								0.255*	0.169*	0.165*	0.159*	0.120*	0.217*
No of admin									0.158*	0.164*	0.165*	0.145*	0.137*
New schools										−0.035*	−0.035*	−0.038*	−0.029*
Lab on computers											0.019*	0.018*	0.015*
Lab on Physics												0.908*	0.824*
Ratio student/teacher													−0.181*
Model improvement													
Deviances based *χ*^2^	–	799.05	7494.7	376003.1	1623483.5	5492.3							
DF	–	2	3	4	5	6							
Value of *p*	–	<0.001	<0.001	<0.001	<0.001	<0.001							

Gray area cells indicate that deviance-based chi-square statistic estimate cannot be computed because the number of parameters between adjacent models is the same.

*Variance reduction by use of a chi-square test based on the difference in the two models’ deviance estimates.

#### 3.2.3. Level-2 predictors

As shown in Table 3, using the same stepwise procedure, school-level predictors were added to the model sequentially eventually keeping significant predictors only. Model improvement is not documented there because the number of degrees of freedom does not change when adding parameters at the between level of the analysis. When the effects of religious schools were tested, results indicated significantly enhanced scores when students were educated in religious compared to non-religious schools by a margin of +5.5 standardized units on the SAAT test. This is a salient difference compared to all other predictors. Two indicators of school size, the number of classes taught, and the number of admin personnel were added in models 8 and 9. Both were significant positive predictors in that larger numbers of classes and administrators were associated with enhanced achievement on the SAAT. Assuming that the two variables represent a proxy of school size, a quadratic effect was tested given recommendations of the relevant literature on the presence of an Inverted-U relationship. Results indicated that the quadratic term of the number of administrators added half percent of predictive variance reflecting very low prediction. Similarly, the quadratic term of the number of classes added 0.002% to the explanation of SAAT performance. Consequently, the theorized quadratic effect of school size on achievement was not supported. Model 10 evaluated the “age” of the school and the negative coefficient (i.e., −0.035) suggested that the older the school the better the students’ achievement. Models 10 and 11 evaluated the contribution of resources such as the presence of computer and physics laboratories. Results indicated that both resources were linked to positive achievement gains (b_Computers_ = 0.019, b_Physics_ = 0.908, *p* < 0.001). Last, model 12 evaluated a ratio variable defining the available teachers as a function of the number of students. Large ratios are suggestive of large numbers of students for small numbers of available teachers. Consequently, smaller ratio values are desirable. Results indicated that the smaller the student-to-teacher ratio, the higher the achievement on the SAAT (b_RatioST_ = −0.181, *p* < 0.001). This finding is particularly more important given that the ratio in Saudi Arabia based on international studies is 11 and was similarly observed in the present study (11.3), which represents a desirable estimate, smaller than most countries in Europe and the rest of the world.

### 3.3. Dependent variable: Number of student absences

**Figure 4:**
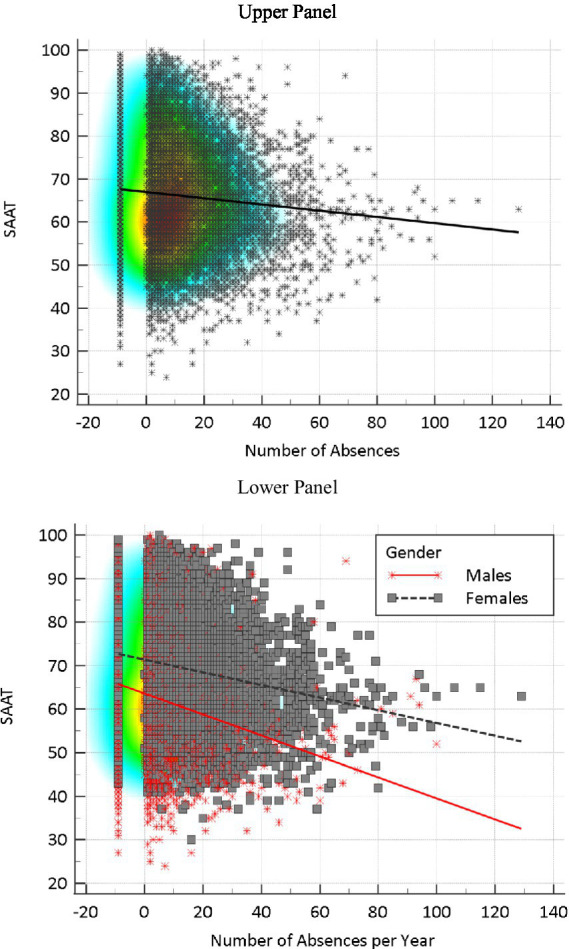
Number of absences as correlates of student achievement in high school using the SAAT measure for the full sample (upper panel) and by gender (lower panel).

Figure 4, upper panel, shows the relationship between the number of absences and student achievement. As shown in the figure, a negative trajectory governed that relationship for the full sample. Thus, the number of absences was consistently associated with lower achievement scores. To address the incongruent finding that females (who were higher achievers compared to males) had also more absences, the prediction of achievement from gender was conducted separately for males and females (see Figure 4, lower panel). As shown in the figure, the number of absences was a significantly more negative predictor of academic achievement in males and less so in females (4.5% versus 5.8% in R^2^). This finding has significant implications on the time spent in school-related activities when absent, which may enlighten the above-mentioned finding.

When predicting the number of students’ absences (see Table 4), positive predictors were being female (*b* = 9.789, *p* < 0.05), and being older (*b* = 1.096, *p* < 0.05). Negative predictors of emitting school absences were having educated parents (*b*_Father_ = −0.604, *p* < 0.05; *b*_Mother_ = −0.633, *p* < 0.05). The gender effect was both significant and salient as on average, females emit almost 10 more absences compared to males.

**Table 4:** Prediction of student number of absences from student level and school level variables.

**Parameter**	**Model1**	**Model2**	**Model3**	**Model4**	**Model5**	**Model6**	**Model7**	**Model8**	**Model 9**	**Model 10**	**Model 11**
Fixed effects											
Intercept of no of absences	13.859*	7.426*	7.652*	10.068*	10.213*	10.225*	9.974*	9.491*	9.499*	9.651*	8.300*
Student level predictor											
Females		9.789*	9.747*	9.354*	10.686*	10.634*	10.769*	9.569*	9.551*	9.362*	9.324*
Age			1.096*	1.313*	1.261*	1.260*	1.240*	1.075*	1.075*	1.073*	1.069*
Father’s Education				−0.604*	−0.288*	−0.288*	−0.301*	−0.429*	−0.429*	−0.428*	−0.427*
Mother’s Education					−0.633*	−0.643*	−0.718*	−0.309*	−0.309*	−0.307	−0.306*
School level predictor											
Religious School						1.966	2.184	2.213*	2.226*	1.989*	2.255*
Private School							4.755*	4.240*	4.295*	4.650*	4.469*
No of Classes								0.196*	0.201*	0.254*	0.178*
Lab on Computers									−0.018*	−0.017*	−0.014*
Lab on Physics										−1.210*	−1.123*
Ratio Student/Teacher											0.151*
Model Improvement											
Deviance based *χ*^2^	–	820.46	8966.89	1579626.8	6358.4						
DF	–	2	3	4	5						
Value of *p*	–	<0.001	<0.001	<0.001	<0.001						

Gray area cells indicate that deviance-based chi-square statistic estimate cannot be computed because the number of parameters between adjacent models is the same. Religious schools were included as a predictor despite being non-significant when introducing school-level predictors. It remained in the model because it gained significance in subsequent steps

*Variance reduction by use of a chi-square test based on the difference in the two models’ deviance estimates.

Among school-level predictors being educated in religious and private schools were significant positive predictors of absences (*b*_Religious_ = 2.213, *p* < 0.05; *b*_Private_ = 4.240, *p* < 0.05, see model 8). Schools with large numbers of classes and with large student-to-teacher ratios were linked to students emitting more absences (*b*_Classes_ = 0.196, *p* < 0.05; *b*_RatioST_ = 0.151, *p* < 0.05); although the number of classes could potentially present a proxy for school size, the respective effect of having large numbers of students failed to reach significance (*b* = 0.001, *p = 0*.391). Last, negative predictors of school absences were the number of computer and physics labs (*b*_Computer_ = −0.014, *p* < 0.05; *b*_Physics_ = −1.123, *p* < 0.05).

## 4. Discussion

The purpose of the present study was to examine predictors of high school students’ achievement from student-level and school-level predictors as fit within Bronfenbrenner’s (1979) ecological theory. Analyses were conducted at the microsystem level (Level-1 in the multilevel model) in which the relationship between student-level characteristics on student achievement were first explored followed by analyses at the mesosystem where the relationship between microsystem variables were explored (Level-2 in the multilevel model). Among student-based predictors, the most important were student gender, and age, the number of absences emitted during the school year, and within home, parental education. Across the nationally standardized achievement measure in Saudi Arabia (i.e., SAAT) results converged along the following conclusions (a) females were higher achievers compared to males (b) older students did worse compared to younger students, (c) more absences were linked to low achievement, (d) and parental education was a positive correlate of student achievement using both father and mother measures.

Among these findings, past international studies were in agreement on the negative role of absences with regard to student achievement (Smerillo et al., 2018) even when levels of absenteeism were saliently lower compared to those in the present study (e.g., in Swedish schools, Karlberg et al., 2022). This finding is particularly worrisome as there is a steady increase in the number of absences over the years. For example, based on the Department of Education data in the United States, the number of absences from high school increased by 6.8% in 2015–2016 compared to 2013–2014. Furthermore, there is a significant distinction between random absenteeism and chronic absenteeism. The latter is defined as emitting 15-day absences per school year (Balfanz and Byrnes, 2012). In the present study, the mean level of absences was 13.86 (intercept in the null multilevel model) and this estimate is very close to what is considered chronic absenteeism using international standards. Thus, the levels of absenteeism are worrisome for students in the Kingdom of Saudi Arabia and more so for female students whose levels of absences are highly elevated (+9.8 absences) compared to those of male students. Given the standardized nature of the SAAT assessment, the negative relationship to students’ absences agrees with earlier findings on standardized measures of math and reading (e.g., Balfanz and Byrnes, 2006; Chang and Romero, 2008; Moonie et al., 2008; Gottfried, 2010).

With regard to parental education, early studies have reported that the positive effects of parent educational levels diminish with the inclusion of other predictors that relate to the family such as income, family structure, and family size (e.g., Stevenson and Baker, 1987; Davis-Kean, 2005; Blondal and Adalbjarnardottir, 2014). The present study’s findings confirm this proposition. Interestingly, past research has mostly investigated the roles of a mother’s education as it is hypothesized that mothers are more actively engaged with children’s education. However, the present study suggests that the father’s education was a saliently more positive correlate of student achievement compared to the mother’s education. This finding is by itself very interesting and contrasts past studies when looking at the weight attributed to each parent’s educational background because a mother’s education has been linked to high expectations by mothers, active engagement, and involvement with their children’s education (Harding et al., 2015) whereas fathers certainly do not follow that trajectory. A hypothesis related to fathers’ correlate maybe income, in that educated fathers, may invest more resources for their children’s education, knowing how important it is (Young and Smith, 1997; Farooq et al., 2011). Obviously, this conclusion needs to be viewed under the lenses of the family structure and family functioning, and the role of parents in Saudi Arabia and may diverge from studies investigating the same phenomenon using European or other samples.

Furthermore, the superior performance of girls over boys has been confirmed recently in a meta-analytic study by O’Dea et al. (2018) across STEM subjects (i.e., science, technology, engineering, and mathematics) although there is still an overrepresentation of boys over girls on these subjects. The findings from the O’Dea et al. study are particularly impressive since they reflect a data synthesis of over 1.6 million students and document salient differences in students’ achievement using standardized effect size measures.

Students’ age is customarily a positive predictor of academic achievement (Navarro et al., 2015), usually termed the relative age effect (RAE, Musch and Grondin, 2001). However, in the present study, a negative effect was observed. Possible explanations are the moderated effects of available time and the need to work, the lack of motivation, etc. Gender was not found to interact with age in producing the low achievement effect for older students.

The mean ratio of students to teachers in the present study using a subsample of the 2016 national data, was 11.134, much smaller compared to international estimates with a mean of 13.9, and close to estimates of most European countries such as the Chech Republic, Spain, Denmark, Hungary, Slovakia, Estonia, Germany, and Switzerland (OECD, 2020) and internationally Russia, Costa Rica, and Japan. Nevertheless, the average estimates of the SA Kingdom, albeit being on the low side, representing a good ratio, was still a significant predictor of academic achievement in that the lower the ratio the higher the academic achievement.

Among school-level predictors, religious schools exerted significantly more salient effects on students’ achievement compared to non-religious schools. This finding may be linked to two important correlates: the overrepresentation of females in religious schools, and the increased demands put forth by these establishments. The number of resources reflected in larger establishments for which there are ample laboratories for science instruction has been linked to positive academic gains (Card and Krueger, 1996; Greenwald et al., 1996; Adeogun and Osifila, 2008; Savasci and Tomul, 2013), although null effects were also observed (Hanushek, 1989, 1997; Wößmann, 2003; Hedges et al., 2016). Thus, a possible explanation is that the presence of ample resources and female students is responsible for the observed relationship between religious schools and enhanced academic achievement (Murillo and Roman, 2011).

In the present study, a large number of classes and administrators seemed to be positively and linearly related to academic achievement despite propositions put forth that the size of the school (usually estimated by the number of students) has a curvilinear relationship to achievement (Crispin, 2016). There are two observations in that regard. First, the magnitude of the relationship was weak and below small estimates of effect size (Cohen, 1992), despite exceeding conventional levels of significance. Second, quadratic effects estimated in addition to linear effects added very little prediction to the linear term again reflecting small effect sizes. Consequently, a conclusion is drawn that, assuming that the number of classes and administrators is a proxy of school size, this relationship is reflected in a positive, albeit weak, covariation. An additional layer of interpretation however is warranted. Administrators do not only add quantitatively to school size but also qualitatively depending on their leadership qualities (Aburizaizah et al., 2019). Specifically, the capacity of administrators such as principals to manipulate teacher incentives, develop a culture of high achievement and high value, or utilize and allocate resources efficiently may be accountable for the positive correlational effect observed in the present study. As Khan (2012) pointed out, the low academic achievement of students may reflect the failure of administrators and policy makers in dealing with specific barriers of the educational system. Our present correlational study design could not disentangle the roles of administrators as instructional leaders (Chapman and Miric, 2009).

### 4.1. Limitations and future directions

The present study is limited for several reasons. First, the stepwise procedure with which predictors were placed in the model was linked to different sample sizes as bivariate type models limit the number of participants who have full data across all predictors. Second, additional predictor variables that have proved to be important in academic achievement were not included (e.g., motivation; see Martin and Lazendic, 2018) in the present study as they were not part of the focus of ETEC and the Ministry of Education in the Saudi Arabia Kingdom and the authors were restricted by what was available from the central authorities that collected the data. Last, given the large sample size involved in the present study, significant effects should be viewed under the lenses of practical significance as otherwise trivial effects can be found to be significant with large sample sizes.

In the future, it would be useful to utilize the present study’s significant predictors of achievement as dependent variables that can be predicted from other predictors. For example, identifying how absences are distributed across the achievement continuum may lead to policies that reduce absences for specific subpopulations (e.g., Heckman and LaFontaine, 2006) using non-linear analytical methodologies (e.g., quantile regression, see Gershenson et al., 2017), rather than relying on crude point estimates. Other researchers have provided similar propositions such as using inverse probability weighted regression analyses (Karaca-Mandic et al., 2012; Smerillo et al., 2018). Furthermore, given the premise from applying Bronfenbrenner’s ecological model, it would be valuable in the future to expand the model through incorporating variables in the exosystem and their interactions with the hierarchies below that It might be good to study the effect of missing data on the performance of the study’s significant predictors of achievement.

## Data availability statement

The raw data supporting the conclusions of this article will be made available by the authors, without undue reservation.

## Ethics statement

The studies involving human participants were reviewed and approved by ETEC. The patients/participants provided their written informed consent to participate in this study.

## Funding

This project was funded by ETEC, Riyadh, Saudi Arabia and ICCTR at BCH.

## Author contributions

GS conceptualized the study and contributed to data analyses and the write-up of the manuscript. AA contributed to data analyses and the write-up of the quantitative sections and also contributed the data for the present illustration. All authors contributed to the article and approved the submitted version.

## Conflict of interest

The authors declare that the research was conducted in the absence of any commercial or financial relationships that could be construed as a potential conflict of interest.

## Publisher’s note

All claims expressed in this article are solely those of the authors and do not necessarily represent those of their affiliated organizations, or those of the publisher, the editors and the reviewers. Any product that may be evaluated in this article, or claim that may be made by its manufacturer, is not guaranteed or endorsed by the publisher.
